# Human impact on the historical change of CO_2 _degassing flux in River Changjiang

**DOI:** 10.1186/1467-4866-8-7

**Published:** 2007-08-09

**Authors:** FuShun Wang, Yuchun Wang, Jing Zhang, Hai Xu, Xiuguo Wei

**Affiliations:** 1 Institute of Applied Radiation, School of Environmental and Chemical Engineering, Shanghai University, Shanghai 201800, China; 2 State Key Laboratory of Environmental Geochemistry, Institute of Geochemistry, Chinese Academy of Sciences, Guiyang 550002, China; 3 Department of water environment, China Institute of Water Resources and Hydroelectric Power Research (IWHR), Beijing 100038, China; 4 State Key Laboratory of Estuarine and Coastal Research, East China Normal University, Shanghai 200062, China

## Abstract

The impact of water quality changes in River Changjiang (formally known as the Yangtze River) on dissolved CO_2 _and silicate concentrations and seasonal carbon flux in the past several decades (1960s–2000) was evaluated, based on monitoring data from hydrographic gauge. It was found that dissolved CO_2 _and silicate in Changjiang decreased dramatically during this decades, as opposed to a marked increase in nutrient (*e.g*. NO_3_^-^) concentrations. Our analyses revealed that dissolved CO_2 _in Changjiang was over-saturated with the atmosphere CO_2_, and its concentration had showed a declining trend since the 1960s, despite that fluvial DIC flux had maintained stable. Analysis results also suggested that the decrease in dissolved CO_2 _concentration was attributed to changes on the riverine trophic level and river damming activities in the Changjiang drainage basin. Due to the economic innovation (*e.g*. agriculture and industry development) across the Changjiang watershed, fertilizers application and river regulations have significantly altered the original state of the river. Its ecosystem and hydrological condition have been evolving toward the "lacustrine/reservoir" autotrophic type prevailing with plankton. Accordingly, average CO_2 _diffusing flux to the atmosphere from the river had been reduced by three-fourth from the 1960s to 1990s, with the flux value being down to 14.2 mol.m^-2^.yr^-1 ^in the 1990s. For a rough estimate, approximately 15.3 Mt of carbon was degassed annually into the atmosphere from the entire Changjiang drainage basin in the 1990s.

## 1. Background

CO_2 _or carbon flux is the major cause in today's global climate change. Rivers, connecting the terrestrial and oceanic ecosystems, plays a unique role in the transportation of weathering products and pollutants from land to ocean. Global fluvial export of carbon is now a well-documented component in the global carbon cycle and is estimated ca. ~10^15 ^g C.yr^-1 ^[1-4]. Although it is relatively small compared with the fluxes at other interfaces (atmosphere-biosphere, atmosphere-ocean), the fluvial carbon flux contributes an important percentage to the regional carbon budget. In addition, carbon exports via rivers are not limited to fluvial discharge. Recently, it has also been found that river systems actively degasses CO_2 _into the atmosphere [2,5,6]. As observed in many studies, concentration of CO_2 _dissolved in rivers, lakes and coastal areas, is higher than its equilibrium concentration relative to CO_2 _in the atmosphere (*i.e*. 350 ppm at present), as a result of biogeochemical process imposed by the thermodynamic equilibrium between the riverine and atmospheric CO_2 _[2,6-15]. Consequently, the excess of CO_2 _can escape to the atmosphere due to physical water- air equilibration.

On the other hand, the increasing anthropogenic activities in river drainage basin, are significantly changing the continuum of land-sea interaction inter-linked by rivers [16,17]. For instance, river damming and eutrophication are widely regarded as the most remarkable and extensive changing events [18,19]. Environment changes (*e.g*. catchment landscape, water quality) can be recorded in riverine carbon inventory, which is closely related to the terrestrial ecosystem [3]. As a result, over-saturation of dissolved CO_2 _in rivers may be misestimated if the estimation is made based on a specific temporal and spatial scale. Hence, we need to look into some historical data from Changjiang for a new understanding in the river geochemistry.

Changjiang is the largest river in Euro-Asian Continent, draining an area of 1.80 × 10^6 ^km^2^. It is ranked third in length (6300 km), fourth for freshwater flow (900 × 10^9 ^m^3^.yr^-1^) and sediment discharge (0.5 × 10^9 ^tons.yr^-1^) in the world [20,21]. During the past several decades (*i.e*. 1960s–1990s), the Changjiang watershed had experienced a significant development of industry and agriculture. Population in the drainage basin had grown over 434,430,000 in 2000, being 1.7 folds that in 1960 [22]. Reported concentration in NO_3_^-^-N, NH_4_^+^-N and NO_2_^-^-N in Changjiang had reached a value that was 5–10 folds higher than the global background values of river water [23]. A recent study suggested that 95% of the nitrate flux of Changjiang could be linked to anthropogenic activities in the 1990s [24], and that DIN in the river was found being doubled in the past two decades [25]. As a result of increases in the nutrients load, the primary productivity (such as diatom) in river can be boosted, by absorbing inorganic nutrients elements (*e.g*. C, N, P, Si) according to Redfield ratio, and then changes the chemical composition of river water. CO_2 _dissolved in the river water can be modified by a disequilibrium of the balance between respiration and photosynthesis in the aquatic ecosystem [26]. It is now well understood that dissolved CO_2 _concentration is a variable rapidly responding to the water quality evolution and trophic status, followed by carbon feedbacks to the atmosphere.

In this study, our main objective was to analyze the influence of human activities on the carbon degassing flux in Changjiang. The historical trend of dissolved CO_2 _concentration in Changjiang was analyzed for the past decades (1960s–2000) based on the monitoring data from select hydrographic station. The change of the inorganic carbon fluvial flux of Changjiang to the East China Sea was also re-evaluated.

## 2. Methods

### 2.1 Area of study

**Figure 1:**
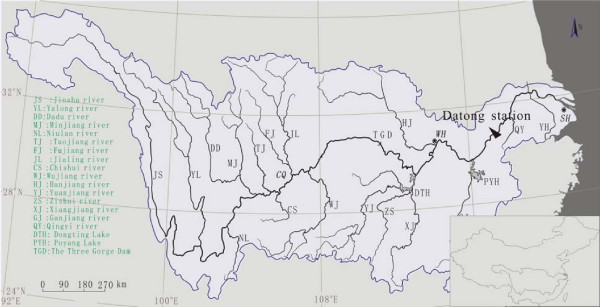
The Changjiang drainage basin and its main tributaries.

The Changjiang drainage basin is situated within 24°30'–35°45'N and 90°33'–122°25'E, draining almost one fifth of the total area of China. The major tributaries in this region include Yalongjiang, Minjiang, Tuojiang, Jialingjiang, Wujiang, Hanshui, Xiangjiang, Yuanjiang, and Ganjiang (Fig. 1). Xiangjiang and Yuanjiang join into the main channel via Lake Dongting and Ganjiang via Lake Poyang. Lake Poyang and Dongting are the two largest freshwater lakes in China [27].

Datong Hydrographic Station (DHS; 117°37'E, 30°46'N as shown in Fig. 1), located 625 km inland from the river mouth on the mainstream, is free from tide influence. There is no major sewage discharge from the industrial and domestic sources nearby. DHS gauges the drainage area of 1.71 × 10^6 ^km^2^, i.e., 95% of the Changjiang watersheds. Therefore, data from DHS have extensively been used to evaluate the variation of water quality of Changjiang and to calculate the seaward flux by the world river in the literatures.

### 2.2. Data source and calculation

DHS was established in 1935. In 1956, the Ministry of Water Resources of China (MWRC) started to systematically monitor the hydrological parameters and water quality along Changjiang, as well as other rivers in China [28]. Data were reported in the internal (unpublished) Hydrological Yearbooks. Monitoring data of water chemistry at DHS over the period 1962 – 1984 presented or used in this study was extracted from the unpublished Hydrological Yearbooks [29]. Data over the period 1985–2000 was officially provided by DHS since data compilation in hydrological yearbooks has been discontinued after 1985. All monitoring data were processed by month, while the annual values were obtained by discharge weighted chemical parameters.

According to station, water was sampled twice per month at DHS. Chemical analyses of the water samples were performed in the laboratory under the authority of the Changjiang Water Resource Commission and following the methods described by Alekin et al. in 1973 [30] and by the American Public Health Association in 1985 [31]. The MWRC also issued its own water quality analytical methods (SL78~94-1994). Specifically, raw water samples were siphoned to determine dissolve CO_2 _concentration using base titration. This method is suitable for surface water analysis, and its standard deviation was reported as 0.105–9.81% when analyzing 17 different kind water samples with dissolved CO_2 _concentration ranging from 2.73–2028 mg/L (SL80-1994). Dissolved water samples were decanted or filtrated through a 0.45-μm membrane from the raw water samples, and analyzed for HCO_3_^- ^(by acid titration, with a standard deviation of 0.71%), and SiO_2 _(by the Hetropoly blue method, with a standard deviation of 1.01%) concentration.

Water samples were filtered through filter papers before 1987 and 0.45-μm filter membranes after 1987 prior to nitrate determination. The filtrates were measured using spectrophotometry with a phenol disulfonic acid before 1987 and UV spectrophotometry after 1987. The phenol method is suitable for measuring concentration ranging from 0.02–2.0 mg/L with a standard deviation of 6.7%, while the UV method is suitable for the concentrations from 0.08–4.0 mg/L with a standard deviation of 1.1%. Both methods are the current Chinese standard for nitrate determination. Therefore, the change of methods had a negligible impact on the accuracy of the data, as has been discussed in detail by Yan et al [21].

Generally, no detailed quality assurance/quality control information is available on historical monitoring data from the annual hydrological reports. Chemical elements in Changjiang are measured at the mg/L scale and the analytical methods are routinely practiced in the national laboratories. Data reported in the yearbooks are therefore considered to be reliable. In fact, Chen et al. (2002) compared the data from another hydrological station on the Changjiang mainstream (Wuhanguan) with data monitored by GEMS/Water Program at the same station. It was found that two sets of data were in good agreement, indicating the reliability of data in the hydrological yearbooks. For detailed reliability analysis of the data in the hydrological yearbooks, see Chen et al., 2000, and Yan, et al., 2003 [21,27].

## 3. Results and Discussion

### 3.1 Hydrographic regime

**Figure 2:**
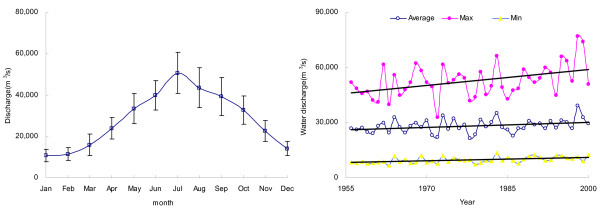
Hydrographic features of Changjiang at Datong. Left:Monthly variation in the water discharge of Changjiang at Datong (average for the period 1956–2000). Right: the annual variation, minimum and maximum water discharge of Changjiang at Datong (from 1956 to 2000). Fit equation for the trend analysis of annual water discharge: y = 87.773x - 145469 r^2 ^= 0.1029 p < 0.05.

Fig. 2 illustrates the seasonal variations of water discharge at DHS in the lower reach of Changjiang, where the maximum monthly water discharge occurs in July and the minimum occurs in January. The magnitude of variation (five folds difference) of water discharge in July and January draws attention to the substantial water input from precipitation in the summer flood season. In addition, as shown in Fig. 2, the annual average of water discharge and minimum discharge were relatively stable, with a slight increase of maximum discharge in the period 1956–2000. It indicates a steady hydrographic feature of Changjiang in the past decades.

### 3.2 Temporal trends of HCO_3_^- ^and dissolved CO_2 _in Changjiang

**Figure 3:**
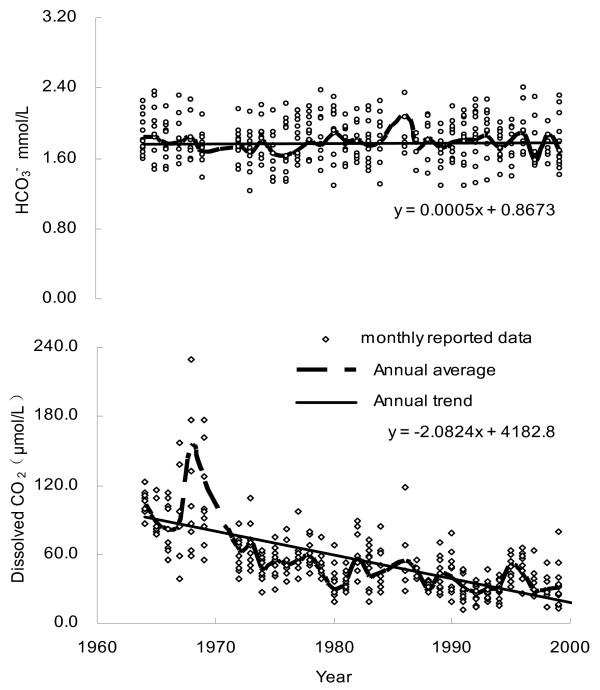
Temporal variations of HCO_3_^- ^and dissolved CO_2 _in Changjiang at DHS (1960s – 2000).

HCO_3_^- ^is mainly derived from the weathering reaction of carbonate and silicate minerals with atmospheric CO_2_, for which atmospheric contribution represents one half of the alkalinity in the case of carbonate weathering and all in the case of silicate weathering. Given the fact that carbonate rocks are well spread in the Changjiang drainage basin, the major element composition of Changjiang and its tributaries is dominated by the HCO_3_^- ^anion. On average, HCO_3_^- ^accounts for 64% (in mg/L units) of the TDS [27]. From Fig. 3, it is clear that there was no clear temporal increase in the HCO_3_^- ^concentrations over the period of 1960s–2000, whereas its inter-annual variation fluctuates between 1.2 and 2.4 mM, with an annual average of 1.7 mM. At the opposite of Changjiang, it was reported that for the Mississippi (the largest river in North America), the export of alkalinity has dramatically increased during the past half-century [32]. The authors ascribed this increase to the change in land cover and heavier rainfall, which resulted in an increase in the rate of chemical weathering. Generally speaking, the rate of long-term chemical weathering involving CO_2 _is mainly correlated with precipitation, stream flow, and temperature [33]. With regard to Changjiang, however, such increase was not observed (Fig. 2, 3), indicating that chemical weathering rate in the Changjiang basin had been maintained a constant for the past decades.

**Figure 4:**
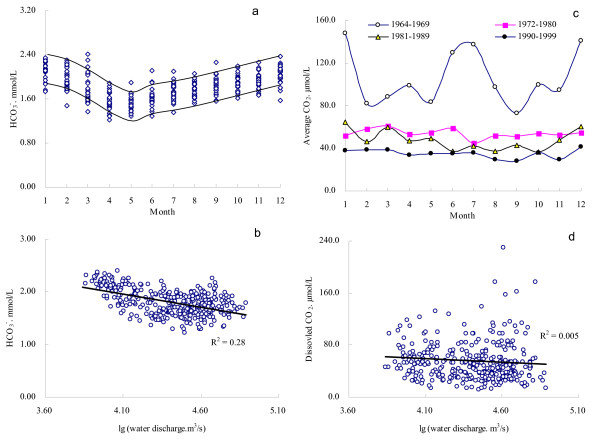
Seasonal variations of HCO_3_^- ^and dissolved CO_2 _in Changjiang at Datong and their relation with water discharge.

To illustrate the seasonal variation, monthly change of the HCO_3_^- ^concentrations in period of 1964–2000 are plotted in Fig. 4. It reveals that HCO_3_^- ^concentrations was measured high in the winter, and low in April through May (Fig. 4a). Although the water discharge of Changjiang showed negative relationship with its HCO_3_^- ^concentration (Fig. 4d), it reached a maximum in July (Fig. 2). This means that dilution was compensated by the effect of increasing dissolution of carbonates as a result of an intensification of soil erosion and elevated *p*CO_2 _during flood season [27].

Data for the past decades (1964s–2000) shows that dissolved CO_2 _concentration of Changjiang has declined dramatically with discharge weighted annual average being reduced from >100 μM in the 1960s to nearly 30 μM by the end of the 1990s (Fig. 3). A peak of dissolved CO_2 _was observed around 1968. Compared with data after the 1970s, monthly concentrations average of dissolved CO_2 _in the 1960s had a higher fluctuation (Fig. 4c). Collectively, water discharge had a less impact on changing the dissolved CO_2 _concentration (Fig. 4d).

In general, dissolved CO_2 _in water will reach equilibrium with the atmosphere CO_2_, as described by the Henry's Law [34]:

CO_2 _(g) + aq = H_2_CO_3_*

K_H _= [H_2_CO_3_*]/*p*CO_2 _(M atm^-1^)

Where, aq stands for the water phase; K_H _is the Henrys Constant; *p*CO_2 _is the partial pressure of CO_2_.

**Figure 5:**
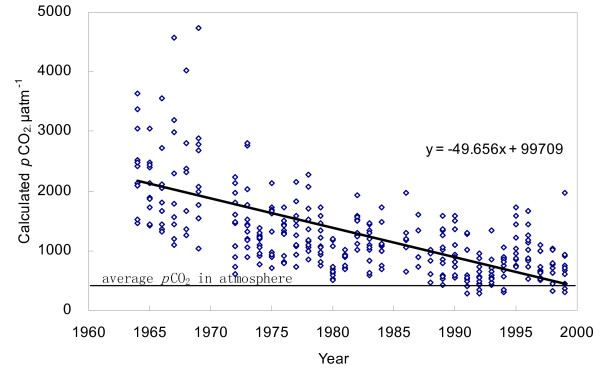
Distribution of *p*CO_2 _in Changjiang at Datong, calculated from dissolved CO_2_.

H_2_CO_3_* is mainly composed of [CO_2_] (*i.e*. dissolved CO_2_) in the natural water phase. In this study, we calculated *p*CO_2 _in Changjiang based on dissolved CO_2 _concentrations according to Equations (1, 2). Results showed that the *p*CO_2 _in the Changjiang riverine system is significantly higher than that of the atmosphere (350 ppm), and it appeared a clear trend of reduction since the 1960s (Fig. 5). In addition, few data in the 1990s have become comparable to the equilibrium concentration of atmospheric CO_2 _(Fig. 5). In fact, it is often the case that CO_2 _is over-saturated in rivers worldwide (Table 1). Compared with rivers listed in Table 1, *p*CO_2 _in Changjiang was 1297 ± 901 ppm, lower than that of other large rivers like Amazon or the Mississippi.

**Table 1:** CO_2 _partial pressure in large rivers in the world

		pCO_2 _μatm		
			
River	Country	Mean	s.d.	Reference
Central Amazon	Brazil	4350	1900	13
Colorado	USA	4295	195	9 #
Columbia	Canada	1123	1175	9 *
Elbe	Germany	4095	1758	9 *
Hudson River Estuary	USA	1125	403	6
Humber	U.K	1500–6000	-	12
Illinois	USA	4419	240	9 #
Lagan River	N. Ireland	2722	1457	55
Main channel of Upper St.Lawrence	Canada	381	93	8
Mississippi	USA	4593	183	9 #
Parana	Brazil	3139	3240	56
Rhone	France	2015	944	9 *
Scheldt estuary	Belgium/Netherlands	5700–9500	-	2
Seine	France	1982	780	9 *
St.Lawrence	Canada	1300	-	11
Upper Jordan	Jordan	2461	608	57
Weser	Germany	4395	2966	9 *
Meech Lake	Canada	6929	8405	26
Changjiang	China	1297	901	This study

#: Originally from Alt, 1993;

*: Originally from Kempe, 1982;

-: Not available.

CO_2 _dissolved in river has sources mainly from the atmosphere CO_2_, respired CO_2 _and dissolution of carbonate [15]. Because *p*CO_2 _in Changjiang was clearly higher than that of the atmosphere, Changjiang should be a potential source for CO_2 _emission to the atmosphere. The basin weathering (such as soil respiration, carbonate dissolution) generally provides a stable input of CO_2 _to river water due to the constant chemical weathering rate. Consequently, in-channel respiration and photosynthesis, i.e. change in water quality, can be responsible for the decline of dissolved CO_2 _in Changjiang.

### 3.3 Decline of dissolved CO_2 _and change of water quality in Changjiang

It is generally accepted that CO_2 _exchange between rivers and atmosphere is an important but still uncertain factor in the global carbon cycle [5,6]. Recent studies estimated that, nearly 0.5 Gt C.yr^-1 ^escapes into the atmosphere from the Amazon basin through air-water CO_2 _exchange, almost an order of magnitude higher than the organic carbon flux from Amazon to the ocean [13]. Since rivers in the world are generally CO_2 _over-saturated (Table 1), the gross emission of CO_2 _from rivers into the atmosphere can be substantial, and it can balance off some important terrestrial carbon sink [35]. On the contrary, the emission flux of CO_2 _from Changjiang had remarkably decreased due to the downtrend of dissolved CO_2 _concentration since the 1960s (Fig. 5). This can greatly affect the estimation of overall CO_2 _exchange flux between Changjiang and the atmosphere, even at the global scale.

#### 3.3.1 Historical change of water quality in Changjiang

In the aquatic ecosystems, two primary biological processes influence *p*CO_2_. They are CO_2 _fixation by photosynthesis and CO_2_emission by respiration [36]. Organic carbon (OC) from the terrestrial ecosystem generally has a steady composition and fixed sources and it is an important carbon source for the aquatic biological processes. This organic carbon, including POC and DOC, usually is of higher proportion in riverine total organic carbon (TOC) [9,37]. However, the anthropogenic activities in the past decades have changed the Changjiang river system. Dam constructions and hydrological regulations were among the most typical activities [38-44]. In addition, the riverine nutrient loads in Changjiang have been greatly increased by the industrial and agricultural activities [45,21]. As a result, the river's pristine status in hydrological condition, nutrients structure and aquatic ecosystem has been shifted to "lacustrine/reservoir" autotrophic type prevailing with planktons [46].

**Figure 6:**
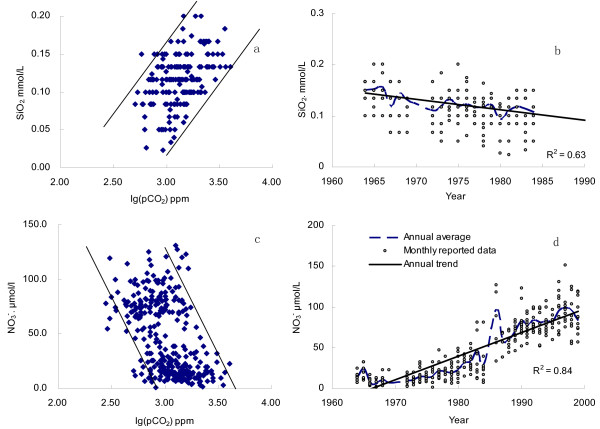
Temporal variation of dissolved SiO_2 _and NO_3_^- ^concentrations in Changjiang at Datong, and their relations with log(*p*CO_2_).

Phytoplankton such as diatoms assimilates dissolved CO_2 _and nutrient elements (e.g. N, P, Si) to synthesize organic components, according to the Redfield Ratio(C:Si:N = 106:16:16) [47,48]. For instance, seriously polluted River Zenne has a lower super-saturation degree of dissolved CO_2 _than unpolluted rivers [2]. With regard to Changjiang, sustaining increase in nutrients concentrations (particularly nitrate) was reported after the 1960s [23]. Increasing in nutrient load stimulates the aquatic photosynthesis. Signatures of C/N and their isotopes composition in the suspend matter also show complex features from both the terrestrial and aquatic ecosystems [49]. Fig. 5 illustrates that the partial pressure of CO_2 _in Changjiang was significantly higher than the atmospheric *p*CO_2_, indicating that carbon source for aquatic photosynthesis was mainly originated from river DIC, not from the atmosphere. During photosynthesis, nutrients are assimilated by phytoplankton (*e.g*. diatoms). Because dissolve silica is derived exclusively from the weathering of silicate in the drainage basin, its concentration (expressed as SiO_2_, data after 1984 are not available) reveals a clear drop in the 1960s–1980s and shows a positive correlation to log(*p*CO_2_) (Fig. 6a,b). However, a dramatic increase of nitrate concentration was observed in the same period, particularly after 1984, with a negative correlation to log(*p*CO_2_) (Fig. 6c,d). This was because that nitrate in Changjiang had long being compensated by the anthropogenic inputs like fertilizers application, similar to rivers around the world [45,50]. In addition to the plentiful nutrient elements introduced by the agricultural activities [21], river damming in the drainage basin also boosted river eutrophication by reducing hydrodynamic condition. More than 40,000 reservoirs had been constructed in the Changjiang basin by 2000, of which over 1100 reservoirs have storage capacities greater than 0.1 × 10^9 ^m^3^. The ecosystems in many reservoirs were facing with the burden of eutrophication [51]. Thus, increasing aquatic photosynthesis will improve the primary productivity, and reduce the dissolved CO_2 _level. In general, the fate of newly formed organic matter in rivers can be (1) buried in lakes and reservoirs as sediments [52]; (2) decomposed in water column; (3) exported into ocean as POC and DOC. Part of the organic matter will be decomposed in estuary, causing the estuarine emission of CO_2 _[2,7].

#### 3.3.2 Temporal trend of CO_2 _diffusion flux

The diffusion flux of CO_2 _can be calculated with the following equation:

Fex=Dz(ceq−c)
 MathType@MTEF@5@5@+=feaafiart1ev1aaatCvAUfKttLearuWrP9MDH5MBPbIqV92AaeXatLxBI9gBaebbnrfifHhDYfgasaacH8akY=wiFfYdH8Gipec8Eeeu0xXdbba9frFj0=OqFfea0dXdd9vqai=hGuQ8kuc9pgc9s8qqaq=dirpe0xb9q8qiLsFr0=vr0=vr0dc8meaabaqaciaacaGaaeqabaqabeGadaaakeaacqWGgbGrdaWgaaWcbaGaemyzauMaemiEaGhabeaakiabg2da9maalaaabaGaemiraqeabaGaemOEaOhaaiabcIcaOiabdogaJnaaBaaaleaacqWGLbqzcqWGXbqCaeqaaOGaeyOeI0Iaem4yamMaeiykaKcaaa@3C98@

Where, *F*_*ex *_is the diffusion flux of CO_2 _between river water and the atmosphere; *D *represents the diffusion coefficient of CO_2 _in the river; *z *is the thickness of boundary layer; *C*_*eq *_in μmol/L is the dissolved CO_2 _concentration in equilibrium with the atmosphere; and *C *in μmol/L stands for the measured dissolved CO_2 _concentration in the water.

Exchanging rate *D/z *of CO_2 _at the water-air interface, can be affected by different factors such as river runoff, turbidity, velocity of flow, wind speed and water depth [2]. It is required to make the estimation of *D/z *a priority, in order to calculate the diffusion flux of CO_2 _in a given system. *D/z *varies from 115 cm/h in a turbulent flow, to 8 cm/h in the rivers without agitation [53]. *D/z *estimates by other researchers are listed in Table 2. Based on the comparison of hydrographic features between Changjiang and world rivers, a *D/z *estimate of 8 cm/h was used for the calculation of CO_2 _diffusion flux from Changjiang.

**Table 2:** *D/z *ratio in world rivers

	*D/z *(cm/h)	Reference
Rhône	15	53
Saône	8–15	53
Amazon mainstream	10	13
Amazon tributaries	5	13
St Lawrence	15	58
Ottawa	4	15
Hudson	4.1	6
Changjiang	8	

Calculation results showed that Changjiang had been degassing CO_2 _into the atmosphere in a declining behavior in the past decades (Table 3). In the 1990s, the diffusing flux of CO_2 _was around 14.2 mol.m^-2^.yr^-1^, similar to that of River Ottawa [15], slightly higher than that of Hudson (5.8–13.5 mol.m^-2^.yr^-1^)[6], and Amazon (10 ± 2.5 mol.m^-2^.yr^-1^) [13]. The CO_2 _degassed from the Amazon basin was estimated almost 13 folds of its fluvial TOC flux (36 Tg C.yr^-1^) or DIC flux (36 Tg C.yr^-1^). Water surface area of the entire Changjiang basin is reported as 9.3 × 10^6 ^ha, or 8.7 × 10^4 ^km^2^. Others reported that it is about 4% of the entire drainage area of the river [54]. In this study, water surface area of 90,000 km^2 ^of the Changjiang drainage basin was taken as an approximate for the flux calculation. Extrapolating across the entire basin, the degassing flux of CO_2 _from Changjiang in the 1990s, was calculated as 15.3 Mt C.yr^-1 ^, about 5 Mt C.yr^-1 ^less than the DIC flux of Changjiang, much less than that of Amazon (0.5 Gt C.yr^-1^). However, this flux of Changjiang in the 1960s was calculated up to 58.8 Mt C.yr^-1 ^(Table 3).

**Table 3:** Historical variation of CO_2_emission flux and DIC fluvial flux in Changjiang

	1960s	1970s	1980s	1990s
***Fex ***mol.m^-2^.yr^-1^	-54.4	-29.7	-21.6	-14.2
CO_2 _degassed from the Changjiang basin. Mt C.yr^-1^	58.8	32.1	23.3	15.3
DIC flux. Mt C.yr^-1^	20.6	17.6	20.4	20.3

-: means CO_2 _is transferred from river into the atmosphere.

***Fex***: CO_2 _flux by evasion into the atmosphere from river

## 4 Conclusion

Our calculation of CO_2 _emission from Changjiang revealed an important pathway for carbon transport from the river to atmosphere. The CO_2 _emission from Changjiang was estimated around 15.3 Mt C.yr^-1 ^in the 1990s, despite that dissolved CO_2 _was less than 5% in DIC. Although CO_2 _degassed from Changjiang had sharply declined by 75% within the past several decades based on our temporal data analyses, it should still be considered as an important source of CO_2 _emission at present.

On the other hand, the nutrient load in Changjiang increased significantly during the past several decades owing to the industrial and agricultural activities and the construction of thousands of reservoirs in the river basin. Consequently, dissolved CO_2 _concentration in the water column had decreased gradually corresponding to changes in the trophic levels of Changjiang over the period of 1960s–2000. As many large rivers around the world are currently facing the increasing anthropogenic impacts similar to Changjiang, re-evaluation of CO_2 _flux between rivers and the atmosphere has become an increasingly important issue in the study of global climate change.

## Authors' contributions

FSW drafted the manuscript, participated in the data calculation and conceived of this study. YCW participated in the data collection. JZ participated in its design and discussion. HX carried out part of the data calculation. XGW helped in the data collection and analysis. All authors have approved this final manuscript.
